# Small Airways Obstruction and Mortality: Findings From the UK Biobank

**DOI:** 10.1016/j.chest.2024.04.016

**Published:** 2024-05-24

**Authors:** Valentina Quintero Santofimio, Ben Knox-Brown, James Potts, Samuel Bartlett-Pestell, Johanna Feary, Andre F.S. Amaral

**Affiliations:** a National Heart and Lung Institute, Imperial College London, London, United Kingdom; b NIHR Imperial Biomedical Research Centre, London, United Kingdom

**Keywords:** FEV_3_/FEV_6_, mortality, small airways obstruction, spirometry

## Abstract

**Background:**

Small airways obstruction (SAO) is common in general populations. It has been associated with respiratory symptoms, cardiometabolic diseases, and progression to COPD over time. Whether SAO predicts mortality is largely unknown.

**Research Question:**

Is spirometry-defined SAO associated with increased mortality?

**Methods:**

Data were analyzed from 252,877 adult participants, aged 40 to 69 years at baseline, in the UK Biobank who had provided good-quality spirometry measurements. SAO was defined as the ratio of the forced expiratory volume in 3 s to the forced expiratory volume in 6 s less than the lower limit of normal. SAO was considered to be isolated if present when the FEV_1_/forced expiratory volume in 6 s ratio was normal (ie, greater than the lower limit of normal). A multivariable Cox regression model was used to assess the association of SAO, and isolated SAO, with all-cause and disease-specific mortality. Sex differences were investigated in these associations, and the primary analysis was repeated, excluding those who ever smoked. All models were adjusted for potential confounders such as sex, BMI, smoking status, smoking pack-years, assessment center, Townsend deprivation index, and ethnicity.

**Results:**

A total of 59,744 participants with SAO were identified, of whom 24,004 had isolated SAO. A total of 5,009 deaths were reported over a median of 12.8 years of follow-up. Participants with SAO had increased all-cause (hazard ratio [HR], 1.31; 95% CI, 1.26-1.36), cardiovascular (HR, 1.39; 95% CI, 1.29-1.51), respiratory (HR, 2.20; 95% CI, 1.92-2.51), and neoplasm (HR, 1.23; 95% CI, 1.17-1.29) mortality risk. These associations were not modified by sex. However, in those who never smoked, only respiratory and cardiovascular mortality risk was associated with SAO. Isolated SAO was also associated with an increased mortality risk (HR, 1.14; 95% CI, 1.07-1.20).

**Interpretation:**

Individuals with SAO have an increased risk of all-cause and disease-specific mortality. Further studies are needed to determine whether SAO causes mortality or is a marker of underlying disease.

## Graphical Abstract

**Figure:**
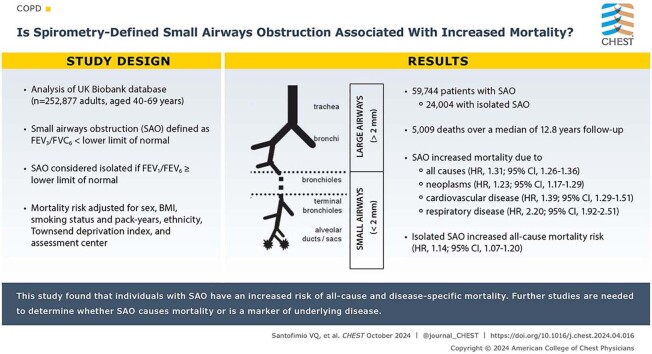

FOR EDITORIAL COMMENT, SEE PAGE 657

**Take-home Points**

**Study Question**: Is small airways obstruction (SAO) associated with an increased mortality risk?

**Results**: In general populations, people with SAO showed greater risk of all-cause and disease-specific mortality compared with people without SAO.

**Interpretation**: The results of this study indicated that SAO is associated with poor survival. However, further studies are needed to understand the role of SAO in influencing mortality outcomes.

Changes in small airways function, particularly small airways obstruction (SAO), may be an early sign of obstructive lung disease.^1^, ^2^, ^3^ Although there is no gold standard to measure SAO, spirometry is the most widely used method.^4^ The mean forced expiratory flow rate between 25% and 75% of the FVC is the most commonly used spirometry parameter for measuring SAO.^5^ However, there has been much debate around its clinical usefulness. This is due to a perceived lack of sensitivity and specificity for assessing the small airways, and its dependence on the accurate measurement of the FVC.^6^^,^^7^ To address these limitations, the forced expiratory volume in 3 s (FEV_3_) to the forced expiratory volume in 6 s (FEV_6_) ratio has been proposed as an alternative to measure SAO. The rationale is that because the FEV_3_ captures a greater fraction of the expired volume than the FEV_1_, it additionally includes air expired from the small airways.^8^ Furthermore, the use of the FEV_6_ removes dependence on the accurate measurement of the FVC.

SAO has been associated with respiratory symptoms,^9^ asthma severity,^10^^,^^11^ and markers of small airways disease on CT imaging,^12^, ^13^, ^14^ as well as future progression to COPD.^1^^,^^2^ The Genetic Epidemiology of COPD (COPDGene) study showed that individuals with abnormal values for the FEV_3_/FEV_6_ ratio have evidence of functional small airways disease, gas trapping, and emphysema on CT imaging.^12^ In addition, the Subpopulations and Intermediate Outcome Measures in COPD Study (SPIROMICS) reported that individuals with SAO are more likely to experience exacerbations and progression to COPD over time, compared with those without SAO.^2^ These findings suggest that SAO defined by using the FEV_3_/FEV_6_ ratio reflects a clinically important abnormality. However, it is currently unknown whether this abnormality confers an increased mortality risk.

Using data from the UK Biobank cohort, the current study investigated the association between SAO using FEV_3_/FEV_6_ and all-cause, respiratory, cardiovascular, and neoplasm mortality. We also aimed to determine whether associations remained in those individuals without established airflow obstruction (ie, with isolated SAO).

## Study Design and Methods

### Study Population

The UK Biobank is a cohort of approximately 0.5 million adults, aged 40 to 69 years at baseline, recruited across England, Scotland, and Wales between 2006 and 2010. Participants provided informed consent, completed questionnaires, and underwent physical measurements. The study was approved by the UK National Research Ethics Service (11/NW/0382, 21/NW/0157).^15^

### Small Airways Obstruction

Spirometry was performed by using a Vitalograph Pneumotrac 68000 Spirometer (Vitalograph Ltd.). A total of two acceptable maneuvers from a maximum of three attempts were required.^16^ Only data that fulfilled previously described quality control criteria^17^ were included in this analysis. SAO was defined as an FEV_3_/FEV_6_ ratio less than the lower limit of normal (LLN). Isolated SAO was defined as an FEV_3_/FEV_6_ ratio less than the LLN, with no evidence of airflow obstruction (FEV_1_/FEV_6_ ≥ LLN).^2^^,^^12^ Reference equations for European American individuals from the third National Health and Nutrition Examination survey were used to derive the LLN for all parameters.^18^ We have previously justified the use of FEV_6_ instead of FVC in the UK Biobank given concerns that the FVC may be underestimated in this population.^19^

### Mortality Data

Mortality data in the UK Biobank were obtained from the National Death Registries. For participants from England and Wales, information on the cause of death was provided by the National Health Service Information Centre; for participants from Scotland, this information was obtained from the National Health Service Central Register. The latest mortality follow-up was on January 31, 2022. The causes of death were coded by using the International Classification of Diseases, 10th Revision. Mortality data were grouped as follows: (1) all-cause mortality, comprising deaths of any cause, excluding external causes (eg, accidental death) of morbidity and mortality (V01-Y89); (2) respiratory mortality (J09-J99); (3) cardiovascular mortality (I05-I89); and (4) neoplasm mortality (C00-C97; D10-D48).^15^

### Statistical Analysis

The mortality risk was estimated from birth to January 2022, with age as time scale for all-cause and cause-specific mortality using a multivariable Cox proportional hazards model.^20^ We built a model for SAO, and isolated SAO, specified as binary variables, and repeated the analysis using FEV_3_/FEV_6_ (percentage) as a continuous proxy for SAO. All models were adjusted for potential confounders: sex (male, female), BMI, smoking status (never smoked, previously smoked, currently smokes), smoking pack-years, assessment center, Townsend deprivation index, and ethnicity (White, other). We also investigated sex differences by running these models separately for male and female participants and adding an interaction term in the models for the product of SAO and sex. A sensitivity analysis limited to those who never smoked was conducted to remove any residual confounding by current or previous smoking.

All analyses were conducted by using the *Survival* and *survminer* packages in R (version 4.1.1.) and RStudio (version 2021.09.0-351).^21^, ^22^, ^23^, ^24^

## Results

### Prevalence of SAO and Isolated SAO

**Figure 1:**
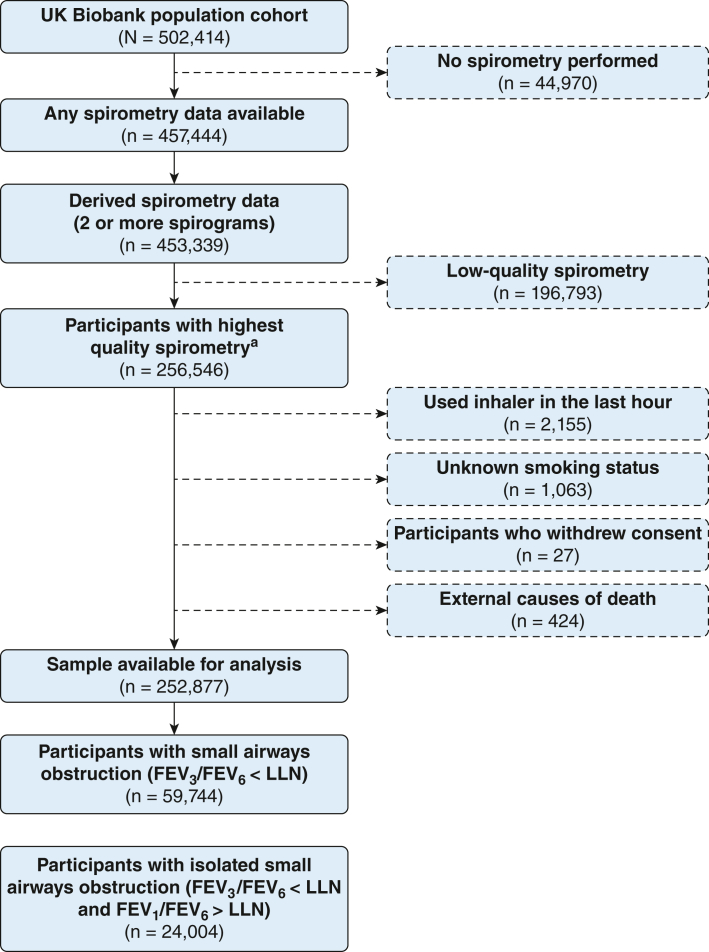
Flowchart of selection of study population. ^a^Highest quality spirometry data: at least two spirograms with no cough, back-extrapolated volume < 5% FVC (or > 5% but < 150 mL), reproducible FEV_1_ and FVC, and a forced expiratory time ≥ 6 s (FEV_6_) on the best curve (curve with highest FEV_1_ and FVC). FEV_3_ = forced expiratory volume in 3 s; LLN = lower limit of normal.

The data of 502,414 UK Biobank participants were obtained; 256,546 participants had high-quality spirometry findings. Participants who had used an inhaler prior to spirometry (n = 2,155), had unknown smoking status (n = 1,063), had withdrawn consent (n = 27), or had external causes of death (n = 424) were excluded from the study. From the final population of 252,877 participants, 59,744 (23.6%) had SAO defined as FEV_3_/FEV_6_ < LLN, of whom 24,004 had isolated SAO (Fig 1).

Among participants with SAO, the majority were female (58.2%), had never smoked (44.7%), and were of White ethnicity (92.1%). Nearly 60% of the participants with SAO had concurrent airflow obstruction (FEV_1_/FEV_6_ < LLN) (Table 1). Participants with isolated SAO had characteristics similar to those with SAO (Table 2). The characteristics of participants without SAO were similar to those of participants with SAO.

**Table 1:** Baseline Characteristics of the Final Population Study

Characteristic	Participants Without SAO			Participants With SAO		
	Female (n = 114,633)	Male (n = 78,500)	Total (N = 193,133)	Female (n = 34,772)	Male (n = 24,972)	Total (N = 59,744)
Age at recruitment, y	56 ± 8	57 ± 7	56 ± 8	56 ± 8	58 ± 8	57 ± 8
Ethnicity						
White	105,105 (91.6%)	72,249 (92.0%)	177,354 (91.8%)	31,915 (91.7%)	23,107 (92.5%)	55,022 (92.1%)
Other	9,528 (8.4%)	6,250 (8.0%)	15,778 (8.2%)	2,857 (8.3%)	1,865 (7.5%)	4,722 (7.9%)
BMI, kg/m^2^	26.3 (23.6 to 29.8)	27.4 (25.2 to 30.1)	26.8 (24.3 to 29.9)	25.3 (22.9 to 28.6)	26.7 (24.3 to 29.4)	25.9 (23.5 to 28.9)
Smoking status						
Never smoked	69,625 (60.7%)	39,959 (50.9%)	109,584 (56.7%)	17,128 (49.2%)	9,606 (38.4%)	26,734 (44.7%)
Currently smokes	7,653 (6.7%)	7,564 (9.6%)	15,217 (7.9%)	5,450 (15.7%)	4,999 (20.1%)	10,449 (17.5%)
Previously smoked	37,355 (32.6%)	30,977 (39.5%)	68,332 (35.4%)	12,194 (35.1%)	10,367 (41.5%)	22,561 (37.8%)
Pack-y of smoking	4.4 ± 10.3	7.4 ± 14.8	5.6 ± 12.4	9.2 ± 15.2	14.7 ± 22.4	11.5 ± 19.0
Townsend deprivation index	–2.3 (–3.7 to 0.1)	–2.3 (–3.7 to 0.1)	–2.3 (–3.7 to 0.1)	–1.9 (–3.6 to 0.7)	–1.9 (–3.6 to 0.9)	–2.0 (–3.6 to 0.8)
FEV_1_, L	2.5 (2.2 to 2.8)	3.4 (3.0 to 3.9)	2.8 (2.3 to 3.3)	2.2 (1.8 to 2.5)	2.9 (2.4 to 3.4)	2.4 (1.9 to 2.9)
FEV_3_, L	2.9 (2.6 to 3.3)	4.1 (3.6 to 4.6)	3.3 (2.8 to 4.0)	2.7 (2.3 to 3.2)	3.7 (3.1 to 4.3)	3.2 (2.5 to 3.7)
FEV_6_, L	3.2 (2.8 to 3.5)	4.4 (3.8 to 4.9)	3.5 (3.0 to 4.3)	3.1 (2.6 to 3.5)	4.2 (3.5 to 4.8)	3.4 (2.8 to 4.1)
FEV_1/_FEV_6_ < LLN	11,334 (9.8%)	8,804 (11.2%)	20,138 (10.4%)	19,808 (57.0%)	15,932 (63.8%)	35,740 (59.8%)

Data are presented as mean ± SD or median (interquartile range), unless otherwise indicated. FEV_3_ = forced expiratory volume in 3 s; FEV_6_ = forced expiratory volume in 6 s; LLN = lower limit of normal; SAO = small airways obstruction.

**Table 2:** Baseline Characteristics of Participants With Isolated SAO in the UK Biobank Cohort (FEV_3_/FEV_6_ < LLN and FEV_1_/FEV_6_ ≥ LLN)

Characteristic	Female (n = 14,964)	Male (n = 9,040)	Total (N = 24,004)
Age at recruitment, y	55 ± 8	56 ± 8	55 ± 8
Ethnicity			
White	13,457 (89.9%)	8,137 (90%)	21,594 (90%)
Other	1,507 (9.1%)	903 (10%)	2,410 (10%)
BMI, kg/m^2^	25.8 (23.4 to 29.1)	27.0 (24.7 to 29.8)	26.3 (23.8 to 29.4)
Smoking status			
Never smoked	8,279 (55.3%)	3,943 (43.5%)	12,222 (50.1%)
Current smokes	1,726 (11.5%)	1,493 (16.6%)	3,219 (13.5%)
Previously smoked	4,959 (33.2%)	3,604 (39.9%)	8,563 (36.4%)
Pack-y of smoking	6.6 ± 12.9	11.4 ± 19.4	8.4 ± 15.8
Townsend deprivation index	–2.1 (–3.6 to 0.6)	–2.0 (–3.6 to 0.8)	–2.0 (–3.6 to 0.7)
FEV_1_, L	2.3 (2.0 to 2.6)	3.2 (2.7 to 3.6)	2.6 (2.2 to 3.1)
FEV_3_, L	2.8 (2.3 to 3.2)	3.8 (3.3 to 4.3)	3.1 (2.6 to 3.7)
FEV_6_, L	3.1 (2.7 to 3.5)	4.2 (3.7 to 4.8)	3.4 (2.9 to 4.1)
FEV_6_ < LLN	2,209 (14.8%)	1,515 (16.8%)	3,724 (15.5%)

Data are presented as mean ± SD or median (interquartile range), unless otherwise indicated. FEV_3_ = forced expiratory volume in 3 s; FEV_6_ = forced expiratory volume in 6 s; HR = hazard ratio; LLN = lower limit of normal; SAO = small airways obstruction.

### Causes of Death

From the final population, 14,842 (5.9%) deaths were reported over a median of 12.8 years of follow-up. One-third of deaths were reported in participants with SAO. The main causes of death were neoplasms (n = 8,289 [55.8%]) followed by cardiovascular diseases (n = 2,921 [19.7%]) and respiratory diseases (n = 937 [6.3%]) (Table 3).

**Table 3:** HRs for All-Cause Mortality and Cause-Specific Mortality in Participants With SAO and Isolated SAO in the UK Biobank Cohort

	All-Cause Mortality			Respiratory Mortality			Cardiovascular Mortality			Neoplasm Mortality		
	Adjusted HR	95% CI	*P* Value	Adjusted HR	95% CI	*P* Value	Adjusted HR	95% CI	*P* Value	Adjusted HR	95% CI	*P* Value
FEV_3_/FEV_6_ < LLN												
Overall	1.31	1.26-1.36	**< .001**	2.20	1.92-2.51	**< .001**	1.39	1.29-1.51	**< .001**	1.23	1.17-1.29	**< .001**
Male participants	1.34	1.28-1.40	**< .001**	2.05	1.72-2.44	**< .001**	1.40	1.27-1.54	**< .001**	1.28	1.19-1.37	**< .001**
Female participants	1.26	1.19-1.33	**< .001**	2.41	1.94-2.98	**< .001**	1.38	1.20-1.59	**< .001**	1.18	1.10-1.26	**< .001**
Never smoked	1.11	1.04-1.19	**.001**	1.49	1.11-1.99	**.008**	1.26	1.09-1.46	**.002**	1.04	0.95-1.13	.40
Isolated FEV_3_/FEV_6_ < LLN												
Overall	1.14	1.07-1.20	**< .001**	1.21	0.94-1.55	.14	1.22	1.08-1.38	**.002**	1.10	1.01-1.19	**.009**
Male participants	1.19	1.10-1.28	**< .001**	1.19	0.80-1.58	.50	1.19	1.02-1.40	**.025**	1.19	1.07-1.32	**.002**
Female participants	1.08	0.98-1.17	.09	1.30	0.89-1.91	.20	1.27	1.04-1.57	**.02**	1.03	0.92-1.14	.60
Never smoked	1.06	0.96-1.16	.20	1.02	0.63-1.67	.90	1.17	0.94-1.45	.20	0.99	0.87-1.12	.90

Boldface identifies *P* values < .05. FEV_3_ = forced expiratory volume in 3 s; FEV_6_ = forced expiratory volume in 6 s; HR = hazard ratio; LLN = lower limit of normal; SAO = small airways obstruction.

Among participants with SAO, there were 5,009 (8.3%) deaths; 2,662 (53.1%) were from neoplasms, 1,038 (20.7%) from cardiovascular diseases, and 474 (9.5%) from respiratory diseases. Among participants with isolated SAO, there were 1,420 (5.9%) deaths, the majority of which were due to neoplasms (n = 793 [55.8%]), followed by cardiovascular diseases (n = 298 [21%]) and respiratory diseases (n = 73 [5.1%]).

### Mortality Associated With SAO

**e-Figure 1:**
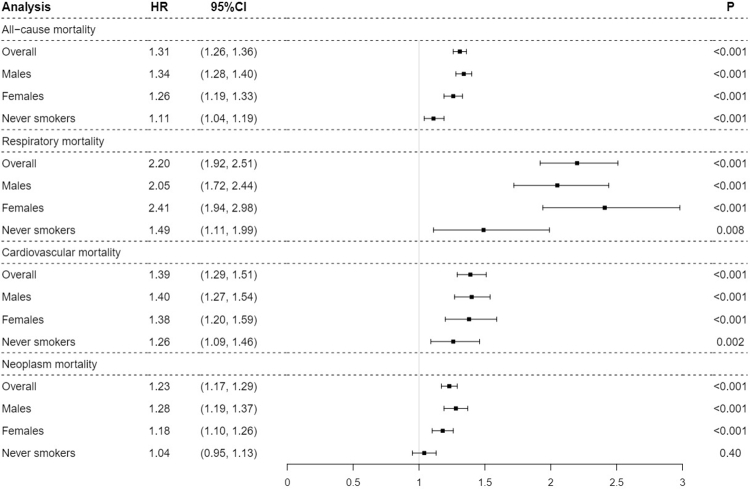
Hazard ratio for all-cause and disease specific mortality associated with SAO

Increased risks of mortality due to all causes (HR, 1.31; 95% CI, 1.26-1.36), neoplasms (HR, 1.23; 95% CI, 1.17-1.29), cardiovascular disease (HR, 1.39; 95% CI, 1.29-1.51), and respiratory diseases (HR, 2.20; 95% CI, 1.92-2.51) were significantly associated with SAO (e-Fig 1, Table 3). There were no material differences in these associations between male and female participants (e-Fig 1, e-Table 1, Table 3). After restricting the analysis to those who never smoked, only the associations with all-cause mortality (HR, 1.11; 95% CI, 1.04-1.19), respiratory mortality (HR, 1.49; 95% CI, 1.11-1.99), and cardiovascular mortality (HR, 1.26; 95% CI, 1.09-1.46) remained significant.

**e-Figure 2:**
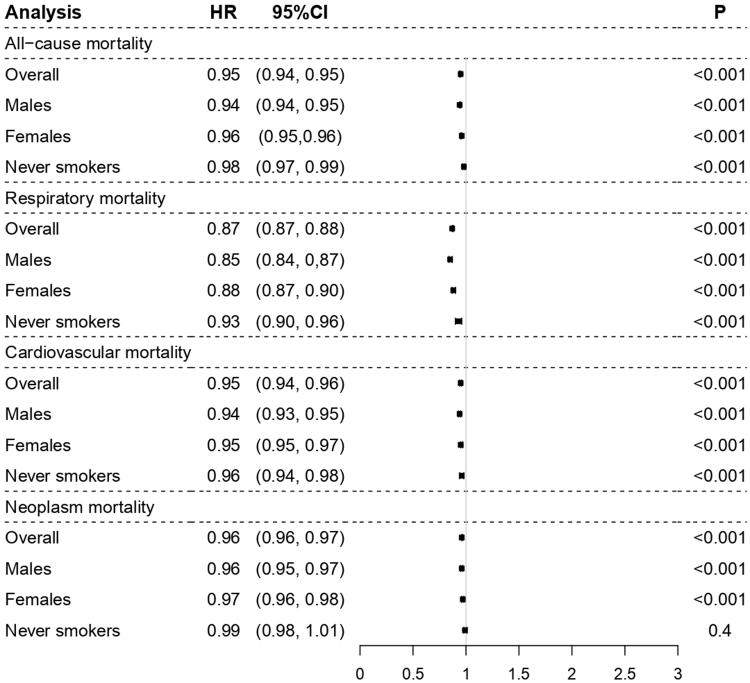
Hazard ratio for all-cause and disease specific mortality per one-percent increase in FEV_3_/FEV_6_

There was a small but statistically significant decrease in all-cause mortality (HR, 0.95; 95% CI, 0.94-0.95), respiratory mortality (HR, 0.87; 95% CI, 0.87-0.88), cardiovascular mortality (HR, 0.95; 95% CI, 0.94-0.96), and neoplasm mortality (HR, 0.96; 95% CI, 0.96-0.97) for each 1% increase in FEV_3_/FEV_6_. In this model, male participants exhibited a slightly lower risk of cause-specific mortality (e-Fig 2, e-Table 2).

### Mortality Associated With Isolated SAO

**e-Figure 3:**
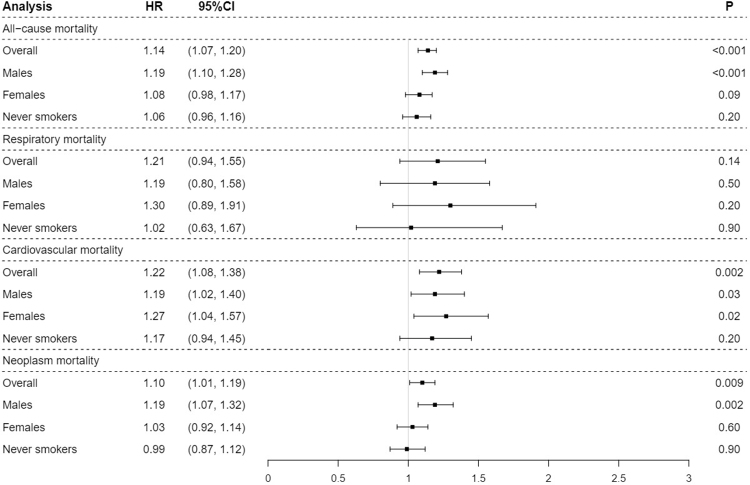
Hazard ratio for all-cause and disease-specific mortality associated with isolated SAO

Isolated SAO was present in approximately 40% of participants with SAO (n = 24,004). An increased risk of all-cause mortality (HR, 1.14; 95% CI, 1.07-1.20), neoplasm mortality (HR, 1.10; 95% CI, 1.01-1.19), and cardiovascular mortality (HR, 1.22; 95% CI, 1.08-1.38) was associated with isolated SAO (Table 3). Respiratory mortality was not associated with isolated SAO (HR, 1.21; 95% CI, 0.94-1.55). There were no material differences in these associations between male and female participants, except for neoplasm mortality, which was slightly higher for male participants (e-Fig 3, e-Table 1, Table 3). There were no associations between mortality and isolated SAO in those who had never smoked (e-Fig 3, Table 3).

## Discussion

To the best of our knowledge, this study is the first to investigate whether SAO defined by using the FEV_3_/FEV_6_ ratio is associated with mortality. We have shown that people with SAO have an increased risk of all-cause, respiratory, cardiovascular, and neoplasm mortality, with the strongest association seen for respiratory-related mortality, in which the risk was more than doubled. We found that the associations did not differ between male and female participants and persisted for both respiratory- and cardiovascular-related mortality when those with a history of tobacco smoking were excluded. Isolated SAO was similarly associated with all-cause and disease-specific mortality risk, although only in male participants.

Overall, 24% of UK Biobank participants had SAO. Limited data are available for direct comparison of the prevalence of SAO in a general population. However, in a previous publication, we estimated that 31% of London residents have SAO using data from the Burden of Obstructive Lung Disease (BOLD) study.^25^ When we examined data from UK Biobank and limited the findings to those recruited in London, the prevalence of SAO was 23.4%. Although these results are not dissimilar, the slight discrepancy could be explained by the use of sampling strategies in the BOLD study to ensure that representative samples of the population were recruited. This was not the case in the UK Biobank study, which is not representative of the UK population with a healthy participant bias.^26^ We also found that 9.5% of UK Biobank participants had isolated SAO. This is similar to that seen for the London site in the BOLD study (13%) but much lower than that seen in the SPIROMICS cohort (17%). This is likely due to the higher proportion of participants who smoked in the SPIROMICS cohort, and participants therefore having a greater risk of obstructive lung disease.^2^ Our finding that isolated SAO is common in the UK Biobank study is important, as these individuals may be at risk of accelerated lung function decline and development of COPD,^1^^,^^2^ highlighting a potential target population for identification of early detection of disease.

The results of this study indicate that individuals with SAO have increased risk of all-cause and respiratory-, cardiovascular-, and neoplasm-related mortality. To our knowledge, only one study has previously investigated similar associations. Costanzo et al^27^ reported results similar to ours when they used longitudinal data from 22,475 participants of the Italian Moli-sani study. They found that individuals with SAO defined by using a forced expiratory flow rate between 25% and 75% of the FVC had a 33% greater risk of all-cause mortality than those without SAO. Similar to the current study, these investigators also found an association between SAO and increased risk of cardiovascular-related mortality. When restricting our analyses to those who never smoked, the association with all-cause and neoplasm-related mortality disappeared, suggesting that residual confounding by tobacco smoking may have accounted for the observed associations for these outcomes. This is likely explained by the close association between tobacco smoking and malignant disease,^28^ as neoplasm-related mortality accounted for > 50% of deaths in those with SAO. Of note, we found that even individuals with isolated SAO had increased risk of all-cause and cardiovascular- and neoplasm-related mortality. No statistical association was observed with respiratory mortality, although the adjusted HR was suggestive of an increased risk. It is possible that this may be due to the small number of deaths in this subgroup of people. In addition, isolated SAO is a mild phenotype that may not be sufficient to drive respiratory mortality alone, and other conditions downstream may be necessary to have a greater impact on mortality. Previous studies have shown that both asthma and COPD are associated not just with respiratory-related mortality but also cardiovascular-related mortality.^29^, ^30^, ^31^ Because SAO is an integral part of the disease process in these respiratory conditions,^32^ our finding that SAO is associated with increased mortality is plausible. However, an increased prevalence of SAO is seen in other conditions in which respiratory function may be affected, including heart failure,^33^ idiopathic pulmonary fibrosis,^34^ and TB.^35^ It is also seen in autoimmune diseases such as rheumatoid arthritis^36^ and inflammatory bowel disease, which themselves can be associated with respiratory disease.^37^ It is thus possible that in these conditions, although established airflow obstruction is not necessarily seen, subtle changes in the airways are sufficient to cause SAO. The mechanisms by which this occurs are likely to be multifactorial; for example, chronic lung disease causing impaired cardiovascular function, increasing the risk of cardiovascular disease^25^ or heart failure causing pulmonary edema and systemic inflammation and resulting in small airways disease. In addition, a history of childhood pneumonia is a risk factor for the development of SAO,^38^ which is important given that childhood infection is a cause of premature mortality in adulthood.^39^

The current findings have several implications. First, we provide evidence that SAO is prevalent in the UK Biobank population, supporting previous research that globally, SAO is more common than airflow obstruction defined by using the FEV_1_/FVC ratio.^25^ Second, our observation that SAO is associated with increased mortality risk provides further evidence that these spirometry parameters are clinically useful and convey important information that may be missed if we rely solely on FEV_1_, FVC, and FEV_1_/FVC to detect spirometric abnormalities. Finally, we have highlighted the potential for measures of SAO to indicate the presence of an underlying disease state or to predict other diseases that may ultimately cause death.

The main strengths of our study are its large sample size and inclusion of only the highest quality spirometry maneuver. Our study also has limitations. First, the UK Biobank cohort is not representative of the general population in the United Kingdom,^26^ which means that our findings may not be generalized to the wider population. Second, we had to exclude a large number of participants with poor-quality spirometry data, reducing statistical power to find associations with cardiovascular- and respiratory-related mortality, in which the number of deaths was low. This may explain why an association was found between cardiovascular-related death and isolated SAO overall but none when stratifying according to sex. Third, the relatively short duration of follow-up time may be insufficient to determine if all those with isolated SAO have increased mortality risk as it could take decades before progressing to more severe airflow obstruction or compromising overall health. In addition, the short duration of follow-up led to a large proportion of participants, who were still alive, being censored at the “end of study” (January 31, 2022). This may have contributed to the low number of deaths observed in the sample. Given the large sample size of this study, it is possible that even weak associations become statistically significant. We therefore emphasize caution in interpreting causality between SAO and mortality, and suggest that further studies in a range of populations should be conducted to confirm or refute these findings.

## Interpretation

The findings of this study indicate that individuals with SAO are at increased risk of all-cause and cardiovascular-, respiratory-, and neoplasm-related mortality. Some of this association is explained by tobacco smoking, particularly for neoplasm-related deaths. However, even those with SAO who have never smoked are at increased risk of premature cardiovascular- and respiratory-related mortality. Further studies are needed to elucidate the causal mechanisms behind these associations, particularly whether SAO is directly causative of mortality or a predictor of downstream disease in the pathway to death.

## Funding/Support

This study was funded by a grant from the 10.13039/501100000856Colt Foundation, United Kingdom (CF/01/21).

## Financial/Nonfinancial Disclosures

None declared.

## Supplementary Data

e-Online Data
