# Supplementary material for: Small Airways Obstruction and Mortality: Findings From the UK Biobank
Source: Chest. 2024 May 24;166(4):712–20. doi: 10.1016/j.chest.2024.04.016 (PMC11492227; doi:10.1016/j.chest.2024.04.016)
Supplement: e-Online Data [file mmc1.docx]

**Supplementary information**

**Title:** Small airways obstruction and mortality: Findings from the UK Biobank

**Valentina Quintero Santofimio^1 *^MSc, Ben Knox-Brown^1*^ PhD, James Potts^1^ BSc, Samuel Bartlett-Pestell^1,2^ MD, Johanna Feary^1,2^ MD, PhD, Andre FS Amaral^1,2^ PhD**

*co-first authors

**Affiliations**

1. National Heart and Lung Institute, Imperial College London, London, UK
2. NIHR Imperial Biomedical Research Centre, London, UK

**Corresponding author:** Valentina Quintero Santofimio, vq20@imperial.ac.uk

**e-Table 1.** Hazard ratios for all-cause mortality and cause-specific mortality for the interaction between SAO and sex in the UK Biobank cohort

|  | **All-cause mortality** | | | **Respiratory mortality** | | | **Cardiovascular mortality** | | | **Neoplasm mortality** | | |
| --- | --- | --- | --- | --- | --- | --- | --- | --- | --- | --- | --- | --- |
| **Interaction** | **Adjusted HR** | **95% CI** | **p-value** | **Adjusted HR** | **95% CI** | **p-value** | **Adjusted HR** | **95% CI** | **p-value** | **Adjusted HR** | **95% CI** | **p-value** |
| FEV_3_/FEV_6_<LLN *Male | 1.03 | 0.96, 1.11 | 0.4 | 0.79 | 0.62, 1.02 | 0.11 | 0.97 | 0.83, 1.14 | 0.7 | 1.07 | 0.97, 1.17 | 0.2 |
| Isolated FEV_3_/FEV_6_<LLN *Male | 1.08 | 0.97, 1.21 | 0.2 | 0.81 | 0.49, 1.34 | 0.4 | 0.92 | 0.71, 1.19 | 0.5 | 1.14 | 0.98, 1.32 | **0.008** |
| FEV_3_FEV_6_*Male | 0.99 | 0.97, 1.01 | 0.07 | 0.97 | 0.87, 0.89 | **0.002** | 0.94 | 0.93, 0.96 | **<0.001** | 0.97 | 0.95, 0.98 | **<0.001** |
| FEV_3_ = Forced expiratory volume in three seconds, FEV_6_ = Forced expiratory volume in six seconds, LLN = Lower limit of normal, HR = Hazard Ratio, CI = Confidence Interval. | | | | | | | | | | | | |

**e-Table 2.** Hazard ratios for all-cause mortality and cause-specific mortality per one-percent increase in FEV_3_/FEV_6_ in the UK Biobank cohort

|  | **All-cause mortality** | | | **Respiratory mortality** | | | **Cardiovascular mortality** | | | **Neoplasm mortality** | | |
| --- | --- | --- | --- | --- | --- | --- | --- | --- | --- | --- | --- | --- |
| **FEV_3_/FEV_6_ (%) continuous** | **Adjusted HR** | **95% CI** | **p-value** | **Adjusted HR** | **95% CI** | **p-value** | **Adjusted HR** | **95% CI** | **p-value** | **Adjusted HR** | **95% CI** | **p-value** |
| Overall | 0.95 | 0.94, 0.95 | **<0.001** | 0.87 | 0.87, 0.88 | **<0.001** | 0.95 | 0.94, 0.96 | **<0.001** | 0.96 | 0.96, 0.97 | **<0.001** |
| Males | 0.94 | 0.94, 0.95 | **<0.001** | 0.85 | 0.84, 0,87 | **<0.001** | 0.94 | 0.93, 0.95 | **<0.001** | 0.96 | 0.95, 0.97 | **<0.001** |
| Females | 0.96 | 0.95, 0.96 | **<0.001** | 0.88 | 0.87, 0.90 | **<0.001** | 0.95 | 0.95, 0.97 | **<0.001** | 0.97 | 0.96, 0.98 | **<0.001** |
| Never smokers | 0.98 | 0.97, 0.99 | **<0.001** | 0.93 | 0.90, 0.96 | **<0.001** | 0.96 | 0.94, 0.98 | **<0.001** | 0.99 | 0.98, 1.01 | 0.4 |
| FEV_3_ = Forced expiratory volume in three seconds, FEV_6_ = Forced expiratory volume in six seconds, LLN = Lower limit of normal, HR = Hazard Ratio, CI = Confidence Interval. | | | | | | | | | | | | |
